# Relationships between self-esteem, self-efficacy, and interoceptive awareness among pole dancers: a network analysis

**DOI:** 10.3389/fpsyg.2025.1716671

**Published:** 2026-01-12

**Authors:** Aleksandra M. Rogowska, Magdalena Mikłuszka, Katarzyna Błońska

**Affiliations:** Faculty of Social Sciences, Institute of Psychology, University of Opole, Opole, Poland

**Keywords:** interoceptive awareness, mind-body connections, physical activity, pole dance, self-efficacy, self-esteem, women

## Abstract

**Introduction:**

Evidence suggests that physical activity is positively associated with interoceptive awareness, self-efficacy, and self-esteem. However, little is known about how these variables interplay within the mind-body connection. This study addresses this gap by examining the relationship between self-esteem, self-efficacy, and interoceptive awareness using network analysis.

**Methods:**

The sample consisted of 263 women participating in a cross-sectional study, aged 18 to 54 years (*M* = 26.00, *SD* = 6.65), including 153 (58.18%) pole dancers. The online survey comprised the Rosenberg Self-Esteem Scale (RSES), the General Self-Efficacy Scale (GSES), and the Multidimensional Assessment of Interoceptive Awareness – Version 2 (MAIA-2), which were used to measure self-esteem, self-efficacy, and interoceptive awareness, respectively.

**Results:**

The Student’s *t*-test showed that women who practice pole dance differ significantly from those who do not in self-efficacy, the overall interoceptive awareness score, and it’s Not-Worrying subscale. The overall network structure was similar in both groups (women who practiced pole dance and those who did not), with minor differences in the strength and number of connections between nodes. Centrality indices revealed the most considerable between-group differences for Body Listening, Non-Distracting, and Non-Worrying. Two primary circuits have been identified, connecting the self-concept (self-esteem and self-efficacy) with the individual dimensions of interoceptive awareness.

**Discussion:**

Evidence suggests that self-efficacy and self-esteem are connected via distinct, specific pathways to interoceptive dimensions within the network of mind-body connections. The differences between groups suggest that participation in pole dance training may significantly impact the network of mind-body interconnections, particularly in relation to two dimensions of interoceptive awareness: Body Listening and Not Worrying. The findings are discussed in the view of the Inference Model, Allostatic Self-Efficacy theory, and Psychosomatic Competence Model.

## 1 Introduction

The growing research on the mind-body connection links the cognitive and behavioral neurosciences to explain the bidirectional relationship in which mental and emotional states profoundly influence physical health, and, conversely, physical conditions affect mental well-being (Littrell, 2008; Georgiev, 2020). The brain communicates with the body by transmitting signals through neural pathways, hormones, and chemicals, affecting everything from digestion to mood. In particular, regular physical activity releases endorphins and serotonin, which can improve mood and reduce symptoms of depression and anxiety (Mikkelsen et al., 2017; Belcher et al., 2021; Mahindru et al., 2023). Numerous studies have supported the notion that enhancing the body-mind connection through approaches such as holistic care, stress management, and mindful movement can lead to better overall health, increased resilience, and well-being (Pelletier, 1992). A balanced mind-body connection is essential for optimal functioning and overall well-being. However, the motivational aspects of this mind-body interaction are unclear, particularly with respect to engaging in a healthy behavior such as physical activity.

Contemporary frameworks for exercise regulation include interoceptive processes in managing physical exertion (Wallman-Jones et al., 2021). Higher-level processes oversee the body’s physiological state to sustain allostasis, while disruptions to these processes affect allostasis. Physical activity directly affects afferent signals processed by the interoceptive system through several mechanisms, including triggering cardiovascular activity and initiating a series of hormonal and metabolic responses (Sellami et al., 2019). Interoceptive mechanisms continuously monitor the body’s physiological state during exercise, maintain allostasis, and guide goal-directed behavior (Craig, 2002; Craig, 2003). Engaging in physical activity can alter the sensory input to the interoceptive system, thereby enhancing the integration of sensory experiences with emotional responses. Nonetheless, the connection between physical activity and top-down regulation is not extensively explored in interoceptive studies. Gaining insight into how interoceptive processes are influenced by physical activity could have important clinical implications for mental health and overall well-being (Wallman-Jones et al., 2021).

Recent evidence suggests that exercise offers a valuable and readily available means to enhance interoceptive processing and promote adaptive behaviors, ultimately leading to a healthy lifestyle and well-being (Barca, 2025). A systematic review and meta-analysis also revealed significant positive associations between physical activity and both dimensions of self-concept: self-efficacy and self-esteem (Babic et al., 2014). These factors significantly influence behavioral control, thereby determining participation in physical activity (Babic et al., 2014). However, the interrelationships between physical activity, interoceptive awareness, and self-concepts, including self-esteem and self-efficacy (as a primary motivational driver), remain unknown. This study fills the gap by exploring the associations between subjective awareness of interoceptive signals from the body and two dimensions of self-concept—self-efficacy and self-esteem—in women who practice pole dance.

Pole dance combines power exercises with sensual movements, producing positive effects on various psychological outcomes, including mental well-being, self-concept, and sexual health (Whitehead and Kurz, 2009; Holland, 2010; Pfeiffer et al., 2023). Research has shown that women participating in pole dancing training received it as an artistic expression that facilitated a sense of liberation and empowerment, enabling them to assert control over their bodies (Whitehead and Kurz, 2009; Nicholas et al., 2018; Nicholas et al., 2024). A qualitative study has demonstrated that pole dancing serves as a creative medium, playing a pivotal role in the lives of women from diverse cultural and socioeconomic backgrounds (Kim et al., 2023a). It promotes body appreciation, self-acceptance, enhances physical health, boosts confidence, provides enjoyment, and fosters social support, thereby increasing self-efficacy, emotional well-being, and overall well-being (Kim et al., 2023a,b). Engaging in pole dancing can be considered an effective self-care strategy that promotes positive body image (Kim et al., 2023a). A satisfied body image is positively related to enjoyment of sexualization and embodiment, while negatively related to self-objectification in women practicing pole dance (Cuccolo, 2021). Pole dancing increases mental well-being, as well as improvements in sexual self-efficacy, sexual anxiety, sexual self-esteem, and body appreciation (Pfeiffer et al., 2023). While correlations among interoceptive awareness, self-efficacy, and self-esteem have been previously identified in a sexual context, the broader associations among these variables have not yet been thoroughly investigated. However, understanding the complex relationship between self-concept and body awareness may have practical implications for the development of future educational programs, mental health interventions, and social interactions.

### 1.1 The current study

Although previous studies have shown associations between self-efficacy and self-esteem, we will, for the first time, examine the relationship between these two dimensions of self-concepts and interoceptive awareness, especially in the context of physical activity, such as specifically feminine pole dance. In particular, the role of self-efficacy in the complex relationships within mind-body connections will be explored for the first time using cross-sectional self-reported measures. The study fills a scientific gap in research on the importance of dance activity for interoception, which may be essential for building self-esteem. This study will examine differences in interoceptive awareness, self-efficacy, and self-esteem between women who practice pole dance and those who do not. Based on previous studies (Babic et al., 2014; Wallman-Jones et al., 2021; Kim et al., 2023a,b; Pfeiffer et al., 2023; Barca, 2025), we hypothesize that women who practice pole dance score higher in interoceptive awareness, self-efficacy, and self-esteem than those who do not dance.

Furthermore, the structure of associations between self-esteem, self-efficacy, and particular dimensions of interoceptive awareness will be explored for the first time using network analysis. The network of interplaying variables will be compared between samples of women who practice and do not practice pole dance to identify the main differences. Using network analysis, we can gain a deeper understanding of the complex, multivariate relationships between self-efficacy, self-esteem, and self-reported interoceptive awareness by visualizing connections between these variables and identifying the unique roles and structural properties of individual variables within a mind-body system. Knowing the mutual connections, we can better plan longitudinal studies and assistance and support programs for people engaging in physical activity, especially to improve their physical health and mental well-being.

## 2 Methods

### 2.1 Study design and procedure

The cross-sectional, online-based study adheres to the ethical standards in psychology, the Declaration of Helsinki, and local regulations. Institutional Research Board (IRB) approved the research protocol (decision KOJBN 31/2023 from 05.12.2023). The sample size was determined using G*Power software (Faul et al., 2007). A minimum sample size of 176 participants (88 in each group) is required to detect the medium effect (*d* = 0.5, *α* = 0.05, 0.95 of power) in the Student’s *t*-test for two independent samples: women who participate in pole dance practice and those who do not. The survey, conducted online via Google Forms, collected data from December 2023 to early March 2024. The survey link was disseminated through Instagram and Facebook groups associated with pole dance and aerial dance, as well as to people engaged in various forms of physical activity.

Additionally, the snowball method was employed by sending the link to women who practiced and those who did not practice these dance techniques. The study’s inclusion criteria required participants to be women and adults, defined as individuals aged 18 years or older. Additionally, membership in the pole or aerial dance group required practicing for at least 6 months. Participants were informed about the study’s purpose, voluntariness, and the guarantee of anonymity. Each respondent received identical instructions and consented to participate; only those who agreed completed the entire questionnaire. A total of 266 participants responded to the invitation; however, two individuals declined participation, and one was under 18 and was excluded from further analyses. The final sample comprised 263 women, demonstrating sufficient power to detect the desired effects.

### 2.2 Participants characteristics

The study included 263 women aged between 18 and 54 (*M* = 26.00, *SD* = 6.65). Among the participants, 153 women (58.18%) engaged in pole dance, whereas 110 (41.82%) reported not participating. In the pole dance group, 41 (26.80%) practiced between half and 1 year, 66 (43.10%) between 1 and 2 years, 30 (19.60%) between 2 and 4 years, and 16 (10.50%) were engaged for five or more years. Participants declared to be engaged also in various forms of physical activity, including 80 persons practicing strength training, 62 in fitness, 55 in yoga, 38 jogging, 36 cycling, 28 in Pilates, 19 swimming, 18 dancing, 18 were engaged in aerobic, 13 in walking, 10 in stretching, 8 in volleyball, and 6 in martial arts. Women engaged in physical activity ranging from 0 to 7 days per week (*M* = 2.79, *SD* = 1.81), with durations spanning from 0 to 240 min on a typical day (*M* = 53.70, *SD* = 44.46).

Most participants resided in medium-sized cities (*n* = 69; 26.24%), while the smallest group lived in small towns (*n* = 31; 11.79%). From the highest education levels, 104 women had a master’s degree (39.54%), 73 a bachelor’s degree (27.80%), 79 secondary education (30.00%), and 3 (1.10%) had elementary education, while 4 (1.50%) had vocational education. Among the participants, 95 individuals (36.10%) were single, while 168 (63.90%) were in a relationship. Furthermore, 73 women (27.80%) were currently engaged in academic studies, 95 (36.10%) were simultaneously pursuing education and employment, 91 (34.60%) reported being employed exclusively, and 4 (1.5%) reported neither working nor studying.

To compare the groups of women who practice and do not practice pole dancing on demographic variables, we used the Mann–Whitney U test for ordinal variables and Pearson’s independence *χ*^2^ test (contingency tables) for bicategorical variables. Both groups do not differ significantly in terms of age, place of residence, or relationship status (Table 1). The group of women practicing pole dance is slightly better educated (*p* = 0.020, RCB = 0.16), more often has the status of a person working than studying (*p* < 0.001, RCB = −0.35) and more often practices physical activity in accordance with World Health Organization (WHO) recommendations (i.e., at least 150 min. Per week; *p* < 0.001, *φ* = 42), exercising significantly more days a week (*p* < 0.001, RCB = 49) and for more minutes each time (*p* < 0.001, RCB = 49) than the group of women not practicing.

**Table 1:** Mann–Whitney *U*-test for differences between women practicing and not practicing pole dance in descriptive variables (*N* = 263).

Descriptive variable	Not practicing pole dance (*n* = 110)		Practicing pole dance (*n* = 153)		*U*	*p*	RBC
	*M/n*	*SD/*%	*M/n*	*SD/*%			
Age	25.35	6.69	26.46	6.60	7,260	0.056	0.14
Place of residence	2.99	1.37	3.06	1.47	8,135	0.638	0.03
Education	3.91	0.80	4.12	1.01	7,084	0.020	0.16
Professional status	2.32	0.69	1.75	0.92	11,383	<0.001	−0.35
Relationship status (single)	47	17.87	48	18.25	3.576^#^	0.059	−0.17
PA WHO recommendations	23	8.75	97	36.88	46.57^#^	<0.001	0.42
Days of PA per week	1.91	1.78	3.42	1.55	4,259	<0.001	0.49
Minutes of PA per week	36.83	41.26	65.82	42.82	4,331	<0.001	0.49

RBC, Rank-Biserial Correlation as an effect size, CI, confidence interval, PA, physical activity, WHO, World Health Organization. # = Pearson’s *χ*^2^ test of independence, with φ statistic as an effect size. Place of Residence was coded: Village = 1, Small town = 2, Medium-sized town = 3, Large city = 4, Huge city = 5. Education was coded: Elementary = 1, Vocational = 2, Secondary = 3, Bachelor degree = 4, Master degree or higher = 5. Current professional status was coded as: neither studying nor working = 0, working = 1, studying = 2, and simultaneously studying and working = 3. Relationship status was coded as: In a relationship = 0; Single = 1. WHO recommendations for PA weekly: PA < 150 min. = 0, PA ≤ 150 min. = 1.

### 2.3 Measures

#### 2.3.1 Self-esteem

The Rosenberg Self-Esteem Scale (RSES) was used to assess self-rated global self-esteem (Rosenberg, 1986). The scale consists of 10 statements evaluated on a four-point scale ranging from 1 (“Strongly agree”) to 4 (“Strongly disagree”). Scores span from 10 to 40, with higher scores indicating elevated levels of self-esteem. The reliability coefficient (Cronbach’s *α*) for the Polish version is reported to range from 0.81 to 0.83 (Łaguna et al., 2007); in the current research, Cronbach’s *α* is 0.90.

#### 2.3.2 Self-efficacy

The Generalized Self-Efficacy Scale (GSES) was used to examine a general sense of perceived self-efficacy (Schwarzer and Jerusalem, 1995), which is considered an individual’s belief in their ability to handle difficult situations, regardless of the specific context. The scale includes 10 statements, with responses rated on a four-point scale from 1 (“Not at all true”) to 4 (“Exactly true”). Higher scores indicate a stronger sense of generalized self-efficacy. Cronbach’s alpha was 0.85 in the Polish adaptation and validation study (Juczyński, 2000) and 0.89 in this study.

#### 2.3.3 Interoceptive awareness

The Multidimensional Assessment of Interoceptive Awareness – Version 2 (MAIA-2) measures multiple dimensions of interoceptive sensitivity, which is understood as a self-reported subjective awareness of bodily sensations (Mehling et al., 2018). The MAIA-2 comprises 37 items arranged in eight scales: Noticing (awareness of body sensations; items 1–4), Not-Distracting (emotional reaction and attentional response to sensations; items 5–10), Not-Worrying (emotional reaction and attentional response to sensations; items 11–15), Attention Regulation (capacity to regulate attention; items 16–22), Emotional Awareness (awareness of mind-body integration; items 23–27), Self-Regulation (awareness of mind-body integration; items 28–31), Body Listening (awareness of mind-body integration; items 32–34), and Trust (trusting body sensations; items 35–37). Participants rate how frequently each statement reflects their typical daily experiences on a 6-point scale from 0 (“Never”) to 5 (“Always”). A higher score (ranging from 0 to 185) indicates a greater awareness of bodily sensations. Cronbach’s alpha ranged from 0.64 to 0.83 in the original study (Mehling et al., 2018) and from 0.55 to 0.91 in the Polish validation (Brytek-Matera and Kozieł, 2015). In the present study sample, Cronbach’s alphas were 0.90, 0.65, 0.81, 0.85, 0.88, 0.85, 0.85, 0.77, and 0.92, for the total score of MAIA-2 and its following scales: Noticing, Not-Distracting, Not-Worrying, Attention Regulation, Emotional Awareness, Self-Regulation, Body Listening, and Trusting, respectively.

#### 2.3.4 Demographic survey

Sociodemographic data were gathered using a metric with 11 closed-ended questions on age (number of years), place of residence (Response options: Village; Small town up to 10,000 inhabitants; Medium-sized town 50,000–150,000 inhabitants; Large city 150,000–500,000 inhabitants; Huge city over 500,000 inhabitants), the highest level of education (Elementary, Vocational, Secondary, Bachelor degree, Master degree or higher), relationship status (Single, In a relationship), Current professional status (studying, simultaneously studying and working, working, neither studying nor working). The subsequent section concentrated on the type of physical activity (open question), the duration of experience in engaging in physical activity (0.5–1 year, 1–2 years, 2–4 years, 5 or more years), the frequency of exercise per week (1–7 days), and the duration of physical activity on a typical day (number of minutes).

### 2.4 Statistical analyses

To assess the parametric properties of self-esteem, self-efficacy, and interoceptive awareness, descriptive statistics were conducted for all scales, including the mean (*M*), standard deviation (*SD*), skewness, and kurtosis. Given the continuous nature of all variables, the large sample size (*N* < 200), and skewness and kurtosis within ±1 for all variables, the normal distribution was considered. Therefore, parametric tests were performed in the following steps. The independent-samples Student’s *t*-test was conducted to test the hypothesis that self-efficacy, self-esteem, and interoceptive awareness differ between women who practice and those who do not practice pole dance. To examine the structure of interactions between self-esteem, self-efficacy, and interoceptive awareness, network analysis (NA) was conducted across samples of women who practiced and did not practice pole dance. The NA was examined using extended Bayesian information criteria and the graphical least absolute deviation and selection operator (EBICglasso) as an estimator. The automatic correlation method was applied, and normalized centrality measures were used for weighted and signed network structures. The NA posits that relationships can be depicted as networks comprising interconnected nodes and links (edges or connections) between them. The direction of associations is indicated by colors (blue signifies a positive association, whereas red denotes negative relationships). The magnitude represents the weighted network between nodes (thicker lines between nodes indicate a stronger relationship). The proximity of nodes also reflects the strength of correlations. Additionally, we employed various centrality indices (such as betweenness, closeness, degree, and expected influence) to pinpoint the most crucial variables and their roles within the network, as well as to evaluate their significance in the model. These statistical analyses were performed using JASP version 0.19.3.0 software for Windows.

## 3 Results

### 3.1 Intergroup differences in self-efficacy, self-esteem, and interoceptive awareness

An independent-samples Student’s *t*-test was performed to assess intergroup differences (Table 2). The sample of women practicing pole dance scored significantly higher in self-efficacy (*p* = 0.001, Cohen’s *d* = −0.41), total score of interoceptive awareness (*p* < 0.050, Cohen’s *d* = −0.26), and its Not-Worrying scale (*p* < 0.050, Cohen’s *d* = −0.32) than those participants who were not practicing. However, the effect size for these differences was small. No statistically significant differences were found for self-efficacy or for the following scales of interoceptive awareness: Noticing, Not-Distracting, Attention Regulation, Emotional Awareness, Self-Regulation, Body Listening, and Trust.

**Table 2:** Student’s *t*-test for differences between women practicing and not practicing pole dance in self-esteem, self-efficacy, and interoceptive awareness (*N* = 263).

Variable	Not practicing pole dance (*n* = 110)		Practicing pole dance (*n* = 153)		*t* (261)	*p*	*d*	95% *CI* for Cohen’s *d*	
	*M*	*SD*	*M*	*SD*				Lower	Upper
Self-esteem	27.40	6.11	29.77	5.50	−3.29	0.001	−0.41	−0.66	−0.16
Self-efficacy	29.33	5.42	30.24	5.20	−1.38	0.168	−0.17	−0.42	0.07
Interoceptive awareness	2.71	0.63	2.87	0.62	−2.10	0.036	−0.26	−0.51	−0.02
Noticing	3.28	0.93	3.37	0.95	−0.71	0.478	−0.09	−0.33	0.16
Not-distracting	2.12	0.99	2.25	0.97	−1.06	0.291	−0.13	−0.38	0.11
Not-worrying	2.04	1.02	2.38	1.09	−2.57	0.011	−0.32	−0.57	−0.08
Attention regulation	2.85	0.96	3.06	0.98	−1.70	0.091	−0.21	−0.46	0.03
Emotional awareness	3.43	1.12	3.63	0.96	−1.56	0.121	−0.19	−0.44	0.05
Self-regulation	2.43	1.12	2.44	1.17	−0.07	0.947	−0.01	−0.25	0.24
Body listening	2.84	1.05	2.79	1.13	0.41	0.681	0.05	−0.19	0.30
Trust	2.95	1.35	3.27	1.37	−1.89	0.060	−0.24	−0.48	0.01

CI, confidence interval. Cohen’s *d* can be interpreted as 0.2 is a small effect, 0.5 is a medium effect, and 0.8 is a large effect.

### 3.2 The structure of associations between self-esteem, self-efficacy, and interoceptive awareness

**Figure 1:**
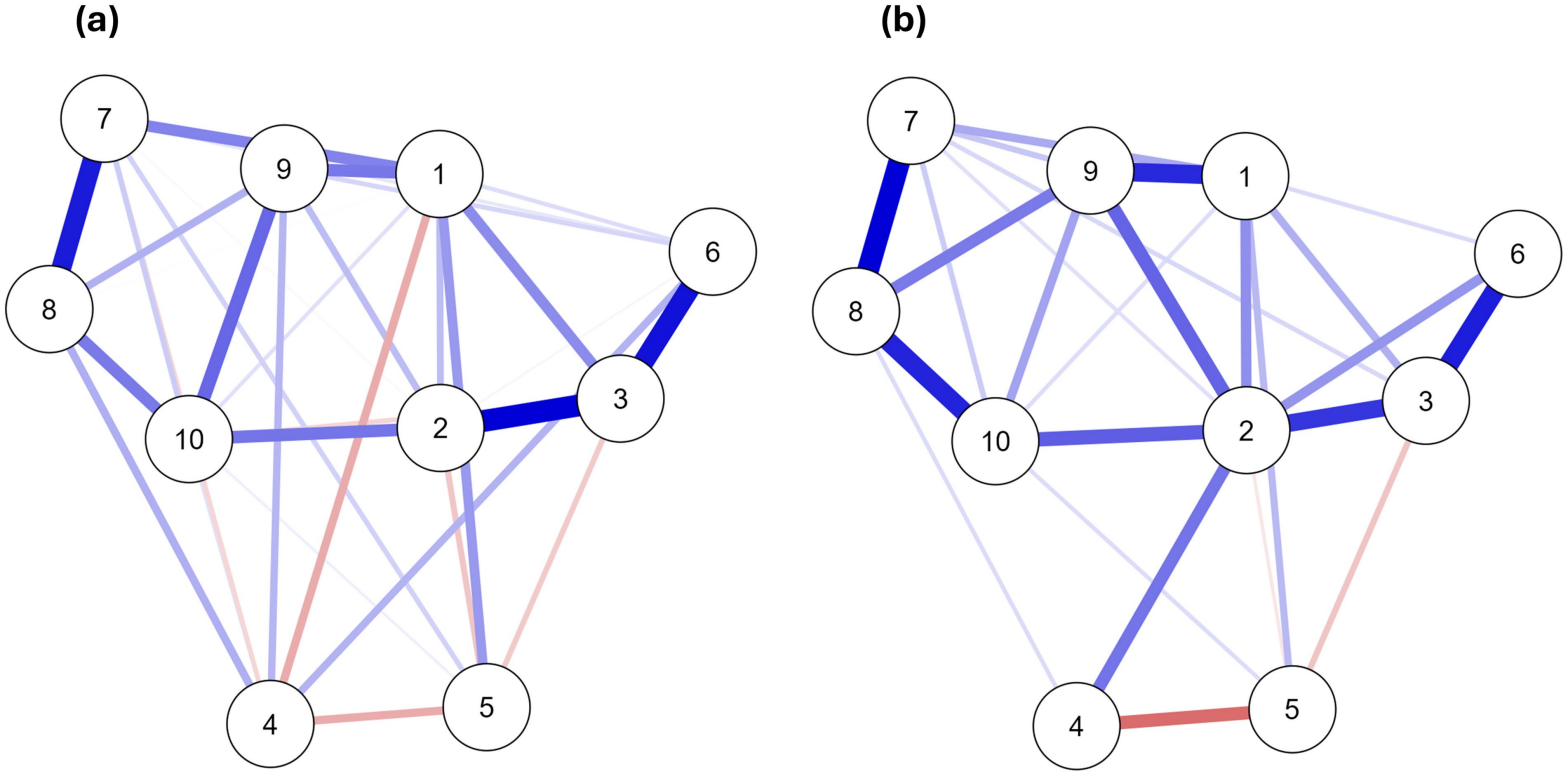
Network plots for associations between self-esteem, self-efficacy, and interoceptive awareness in **(a)** a sample of women not practicing pole dance, and **(b)** women practicing pole dance. 1 = Attention regulation, 2 = Body listening, 3 = Emotional awareness, 4 = Not distracting, 5 = Not worrying, 6 = Noticing, 7 = Self-efficacy, 8 = Self-esteem, 9 = Self-regulation, 10 = Trusting. The blue line indicates positive associations, and the red line indicates negative associations between nodes.

**Figure 2:**
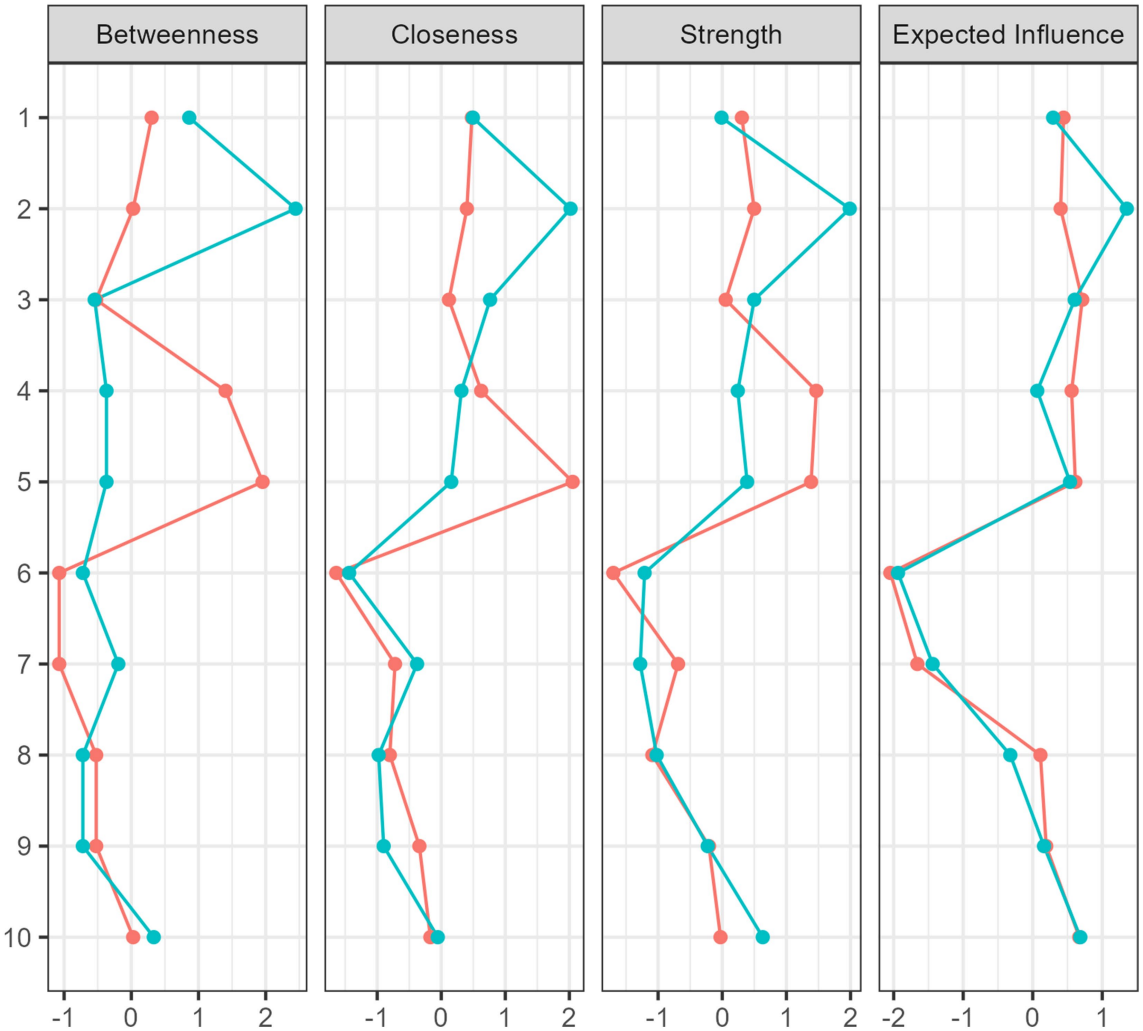
Centrality plots for associations between self-esteem, self-efficacy, and interoceptive awareness in the sample of women who do not practice pole dance (red line) and those who practice pole dance (blue line). 1 = Attention regulation, 2 = Body listening, 3 = Emotional awareness, 4 = Not distracting, 5 = Not worrying, 6 = Noticing, 7 = Self-efficacy, 8 = Self-esteem, 9 = Self-regulation, 10 = Trusting.

The structure of interplay between self-esteem, self-efficacy, and interoceptive awareness was explored using network analysis (Figures 1, 2). The overall structure is similar for both groups, with small differences in strength and the number of connections between nodes (Figure 1). Furthermore, centrality indices showed the most remarkable intergroup differences in Body Listening, Not Distracting, and Not Worrying (Figure 2). Betweenness centrality indices indicated that Body Listening had the greatest influence on the whole network in the pole dancer sample, while Not Distracting and Not Worrying were more critical in the non-pole dancer sample. For both samples, the greatest expected influence was Attention Regulation, Body Listening, Emotional Awareness, Not Distracting, and Not Worrying. In contrast, Noticing, Self-Efficacy, and Self-Esteem demonstrated negative relationships with the other nodes in the network, as measured by betweenness, closeness, and strength (Figure 2).

## 4 Discussion

### 4.1 Differences in self-esteem, self-efficacy, and interoceptive awareness between women who practice and do not practice pole dance

The study was conducted among women who practiced and did not practice pole dance to examine intergroup differences in self-concept and interoceptive awareness. Based on previous studies (Babic et al., 2014; Cuccolo, 2021; Kim et al., 2023a,b; Pfeiffer et al., 2023; Barca, 2025), we assumed significant intergroup differences, suggesting higher scores in all variables among women who practice pole dance, compared to those who do not. These assumptions were partially supported in this study. The Student’s *t*-test revealed intergroup differences in self-esteem and interoceptive awareness, as measured by the total score and the Not-Worrying subscale of the MAIA-2. Consistent with the hypothesis, women who engage in pole dancing exhibited higher scores on these variables than those who do not. Still, the effect size for these findings was small. However, participation in pole dancing does not appear to significantly influence self-efficacy or most dimensions of interoceptive sensitivity.

A narrative review suggests that engaging in physical activity offers a valuable and accessible approach to enhancing interoceptive processing (Barca, 2025). Interoception is the sensory system that enables individuals to perceive their physiological conditions, including autonomic sensations such as pain and touch (Craig, 2002; Craig, 2003). Mehling et al. (2009) describe body awareness as the conscious perception of bodily states, processes, and actions, influenced by mental activities. Wallman-Jones et al. (2021) argue that interoceptive processes regulate effort during physical activity, thereby facilitating the processing of internal body signals. Christensen et al. (2018) found that dancers exhibited higher interoceptive accuracy, measured by heartbeat detection, than non-dancing women. Qualitative studies indicate that women who participate in pole dance report a strong sense of control over their bodies (Whitehead and Kurz, 2009; Nicholas et al., 2018, 2024).

Furthermore, previous studies have shown that athletes scored higher on various interoceptive awareness scales compared to non-athletes (Rogowska et al., 2023; Seabury et al., 2023). Seabury et al. (2023) examined differences in interoceptive awareness among sprinters, long-distance runners, and non-athletes using the MAIA questionnaire and two tasks (heartbeat counting and detection in silence and crowd noise). Results indicated that sprinters and long-distance runners scored higher on the Trusting, Attention Regulation, and Self-Regulation scales and demonstrated superior interoceptive skills. Other research has demonstrated that athletes scored higher than non-athletes in Noticing, Attention Regulation, Emotion Awareness, Self-Regulation, Body Listening, Trusting, and the overall score of the shortened MAIA-2 questionnaire, indicating that regular physical activity enhances interoceptive abilities (Rogowska et al., 2023). In contrast to the present study, the Not-Worrying scale did not differ between athletes and non-athletes (Rogowska et al., 2023). These discrepancies between the present and previous studies may be related to the specific discipline, the level of competition versus recreational engagement in physical activity, and also the years of sports experience. Future studies should focus on comparing interoceptive awareness across various sports disciplines and competition levels (local, regional, national, international) while controlling for years of competition experience. Our study suggests that engaging in pole dance has only a negligible effect on the cumulative score of all dimensions of interoceptive awareness. Given the tendency towards grooving, we can assume that greater pole dance experience may contribute to a greater distance between groups in interoceptive awareness. To test this speculation in our research, we conducted an additional Spearman correlation analysis, which showed that only the Not Worrying scale of MAIA-2 was positively associated with pole-dancing practice duration (*Rho* = 0.15, *p* = 0.015). However, given that the vast majority of women had been pole dancing for 1–2 years, these results should be replicated in a larger, more experienced group of dancers.

Previous studies suggest positive relationships between physical activity engagementnd both dimensions of self-concept (Babic et al., 2014). Our study confirmed that practicing pole dance increases self-esteem, but has no effect on self-efficacy among women. Previous qualitative research has demonstrated that participation in pole dance training enhances various aspects of self-concept, including self-acceptance, body appreciation, positive body image, self-confidence, and self-efficacy (Kim et al., 2023a,b). Additionally, pole dance has been shown to improve body appreciation and both dimensions of self-concept: self-efficacy and self-esteem, particularly in the context of sexuality (Pfeiffer et al., 2023). Research indicates that increased physical activity is positively correlated with self-esteem, body image, and self-efficacy amongniversity students (Doymaz et al., 2024). The Exercise and Self-Esteem Model (EXSEM) provides a framework for understanding these relationships, suggesting that physical activity influences self-esteem through mediating factors such as body appreciation and perceptions of physical fitness (Sonstroem and Morgan, 1989; Jankauskiene and Baceviciene, 2022). This study appears to align with the EXSEM, confirming a large body of previous research. The absence of significant differences in self-esteem between women who engage in pole dance and those who do not may be attributed to the unique characteristics of this particular discipline. To elucidate this discrepancy, further research is warranted that compares various disciplines, considering both competitive and recreational participation. However, it is essential to note that although power analysis indicated that the sample of 263 people would be adequate to detect a medium effect size, the results indicated small effect sizes for the between-group differences. Despite statistical significance, the practical differences between groups were marginal. Unfortunately, we cannot definitively determine whether the non-pole-dancing group engaged in other forms of physical activity or sports, as we did not examine this aspect. To address this issue, future research should compare pole dancers with individuals engaged in other forms of physical activity to determine whether pole dancing contributes explicitly to differences in self-esteem and interoceptive awareness, or whether other forms of physical activity would have a similar effect on these variables.

### 4.2 Network analysis for associations between self-esteem, self-efficacy, and interoceptive awareness

This study examined the mind-body connections, focusing on the intricate relationships between interoceptive awareness, self-efficacy, and self-esteem. Network analysis revealed that self-esteem is positively, closely, and strongly associated with self-efficacy, regardless of whether the sample consisted of women who practiced pole dance or did not. Self-efficacy and self-esteem are two dimensions of self-concept, which refers to the comprehensive beliefs, attitudes, and perceptions that an individual has of their own identity, personality traits, skills, past experiences, and behavioral characteristics, essentially answering the question, “Who am I?” (Ayduk et al., 2009; Leflot et al., 2010). While self-efficacy and self-esteem are closely related, they are distinct constructs (Solanki, 2022; Lee and Lee, 2024). Self-efficacy refers to an individual’s belief in their ability to perform specific tasks or behaviors (Bandura, 1977; Bandura, 1986; Resnick, 2018). In contrast, self-esteem relates to an individual’s overall sense of self-worth and value (Rosenberg, 1986; Cameron and Granger, 2019). Research consistently shows a positive relationship between both dimensions of self-concept: self-efficacy and self-esteem (Blake and Rust, 2002; Lane et al., 2004; Hajloo, 2014; Jaaffar et al., 2019; Noorollahi, 2021; Solanki, 2022; Zhang et al., 2022; Cao and Liu, 2024). Individuals with higher self-efficacy tend to have higher self-esteem, and vice versa. Both self-efficacy and self-esteem have been linked to various psychological outcomes in previous studies, like procrastination, career identity, or decision-making, academic performance, workload, and well-being (Lane et al., 2004; Hajloo, 2014; Tan et al., 2015; Bonsaksen et al., 2017; Molero et al., 2018; Noorollahi, 2021; Pignault et al., 2023; Cao and Liu, 2024; Coelho et al., 2024; Lee and Lee, 2024).

Some studies suggest that self-efficacy can predict self-esteem levels (Tan et al., 2015; Coelho et al., 2024). This implies that as a person’s belief in their ability to accomplish tasks (self-efficacy) increases, their overall sense of self-worth (self-esteem) is likely to improve as well. However, other studies have found that self-esteem can influence changes in self-efficacy (Pignault et al., 2023; Cao and Liu, 2024). It suggests a potentially reciprocal relationship between the two constructs. Indeed, a longitudinal study found the reciprocal relationship between self-esteem and general self-efficacy among students from both the elite and non-elite universities in China (Zhang et al., 2022). Among students at prestigious universities, self-esteem and overall self-efficacy mutually enhanced each other from first to third year. In contrast, for students at non-prestigious universities, self-esteem had a unidirectional influence on their later general self-efficacy from their second year to their final year (Zhang et al., 2022).

**Figure 3:**
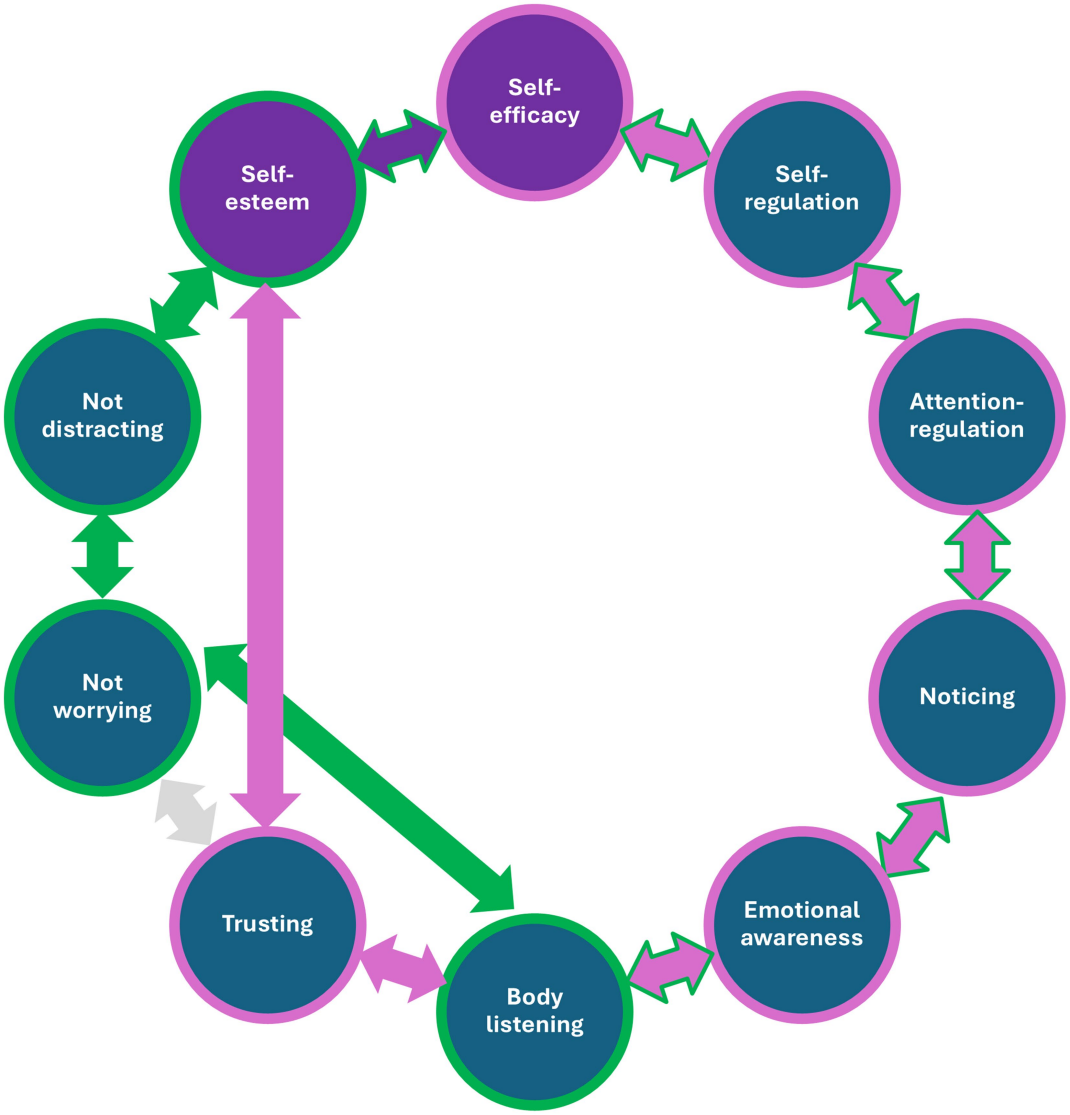
Model of mind-body connections linking self-efficacy and self-esteem with interoceptive awareness. The first circuit is marked with pink arrows, the second with green arrows. Purple circles indicate self-esteem and self-efficacy, while blue circles indicate all dimensions of interoceptive awareness.

Considering the associations between self-concept and specific scales of interoceptive awareness, self-esteem was strongly and closely related to trusting one’s body signals in this study (Figure 3). The first chain of mind-body connections leads from self-esteem, via Trusting, Body Listening, and Emotional Awareness, to Noticing (see pink arrows in Figure 3). The second chain of mind-body connections begins with self-efficacy and leads to noticing through self-regulation and Attention Regulation (see blue arrows in Figure 3). The first circuit links self-efficacy and self-esteem via both chains of interoceptive dimensions, with the reversal occurring in the Noticing subscale.

Self-esteem is also directly linked to Not-Distracting, which, in turn, mediates relationships with Not-Worrying and extends to both Body Listening and Emotional Awareness (see the green arrows in Figure 3). However, this second circuit is more distant and weaker than the first (linking the blue and pink arrows in the Noticing scale). The structure of interconnections between self-efficacy, self-esteem, and specific dimensions of interoceptive awareness is similar in both samples of women who practice and do not practice pole dance. However, the connections within a pattern are stronger in women who practice pole dancing than in those who do not. In particular, Body Listening has stronger connections and more links with other dimensions of interoception in the sample of women practicing pole dance than in the non-practicing group, playing a crucial role in their network structure.

Analysis of centrality indices in NA (betweenness, closeness, strength, and expected influence) revealed that the greatest differences between samples were consistently observed in Body Listening, suggesting that engagement in pole dance benefits mind-body connection. In contrast, Emotional Awareness and Attention Regulation were more important for the sample of women who did not practice pole dance, suggesting various patterns of interplay between interoceptive awareness and both dimensions of self-concept, namely self-efficacy and self-esteem. Two interoceptive dimensions, Not-Worrying and Not-Distracting, have the greatest negative influence on the network structure in both groups. This result suggests that preventive and intervention programs should focus on reducing negative thinking, ruminations, and negative emotions, particularly anxiety, to enhance mind-body connections and overall well-being.

Interoceptive awareness plays a crucial role in influencing self-efficacy by enhancing self-regulation, emotional regulation, and performance in specific domains. Interoceptive awareness and self-efficacy shared numerous characteristics related to self-regulated processes, which may explain their association. Interoceptive awareness guides adaptive behavior by providing ongoing information about body signals, which is essential for rational decision-making and empathy. In physical activities like climbing, psychological training interventions that improve interoceptive awareness also enhance self-confidence and climbing ability (Garrido-Palomino and España-Romero, 2023). Furthermore, baseline interoceptive awareness can predict the outcomes of interventions designed to improve self-efficacy. For instance, in the context of social anxiety disorder, initial levels of interoceptive awareness were found to predict the effectiveness of cognitive behavioral therapy in improving self-efficacy (Schmalbach and Petrowski, 2025). This suggests that individuals with higher interoceptive awareness may respond more effectively to interventions designed to enhance self-efficacy.

Better awareness of internal bodily signals can help individuals regulate their emotions and behaviors more effectively, thereby boosting their confidence in their abilities. Enhanced interoceptive awareness is associated with improved emotional regulation, a crucial component of self-efficacy. Interoceptive awareness is positively associated with emotional clarity and goal clarity, which are essential for self-efficacy (Gross et al., 2025). Individuals with higher interoceptive awareness can better understand and regulate their emotions, contributing to a stronger sense of self-efficacy (Mahler et al., 2024; Gross et al., 2025). Effective self-regulation, influenced by interoceptive awareness, is crucial for maintaining emotional stability and achieving goals, thereby enhancing self-efficacy and self-esteem (Cheung et al., 2024; Mahler et al., 2024). Studies have demonstrated that interventions targeting interoceptive awareness can lead to significant improvements in emotional regulation (Mahler et al., 2024; Schmalbach and Petrowski, 2025). In particular, better management of stress and anxiety supports higher self-efficacy (Mahler et al., 2024; Schmalbach and Petrowski, 2025).

Improved interoceptive awareness, achieved through interventions such as mindfulness, enhances self-regulation and emotional stability, thereby both directly and indirectly boosting self-efficacy and contributing to self-esteem (Bornemann et al., 2014; Matiz et al., 2020). For example, mindfulness interventions, based on body scans and breath meditation that enhance interoceptive awareness, have also been linked to increased self-efficacy in various contexts, including academic settings and physical performance (Bornemann et al., 2014; Fagioli et al., 2023; Cheung et al., 2024; Schoeller et al., 2024). Numerous studies have demonstrated that mindfulness-based interventions (MBIs) improve interoceptive awareness by enhancing self-efficacy and self-esteem (Britton et al., 2021).

Current frameworks, such as the Interoceptive Inference Model (Seth, 2013) and the concept of Allostatic Self-Efficacy (Stephan et al., 2016; Krupnik, 2024), provide further theoretical explanations for the present results. These models suggest that accurate interoception (awareness of the body’s internal states), combined with the ability to interpret bodily signals as a sign of effective self-regulation, fosters a sense of competence and control, thereby increasing self-efficacy, which in turn supports higher self-esteem. The Active Inference framework (Friston et al., 2009) provides a foundation for such connections by describing how the brain utilizes interoceptive signals to build models of the self and the world (Yang et al., 2023; Pezzulo et al., 2024a,b). The concept of active inference suggests that conscious behavior relies on the brain’s inherent use of internal models to anticipate, infer, and guide actions (Pezzulo et al., 2024a,b). High interoceptive sensitivity may lead to a more accurate self-model, which could influence self-esteem and self-efficacy by providing reliable information for self-regulation and decision-making (Khoury et al., 2018; Chester et al., 2024; Krupnik, 2024; Barca, 2025).

Self-regulation and the feedback loop play crucial roles in the relationships among these variables. The Psychosomatic Competence Model (Fazekas et al., 2010; Fazekas et al., 2022a,b) conceptualizes interoceptive awareness as a core component of self-regulation and health behavior. The model posits a feedback loop between interoception and self-regulation, monitored by cognitive factors, and is supported by reported correlations between interoceptive awareness, mentalization, and self-efficacy. Schwerdtfeger et al. (2019) and Cella et al. (2019) provided empirical support for the role of interoceptive awareness in moderating or mediating the effects of cognitive reappraisal and self-efficacy on psychological and behavioral outcomes, such as self-esteem, heart rate variability, and impulse regulation. Self-efficacy is theorized to be strengthened by accurate interoceptive awareness, as individuals who can interpret bodily cues are better able to regulate emotions and behaviors, leading to higher self-esteem (Cella et al., 2019; Benau, 2023; Chammas et al., 2024). Positive feedback loops contribute to successful self-regulation (e.g., healthy eating) and enhance self-efficacy and self-esteem, which, in turn, further improve interoceptive awareness (Chammas et al., 2024). These studies highlight the significance of interoceptive awareness in promoting adaptive self-regulation and psychological well-being, as noted by the authors.

### 4.3 The limitations of the present study and future directions

The present study is subject to certain limitations that restrict the generalizability of its findings. The sample was conventionally selected and recruited online through social media and a private mailing list, which may have introduced bias and measurement error. Future research should address this limitation by directly engaging with pole dance practitioners across various sports clubs. Additionally, the control groups should be broadened to encompass other sports disciplines and recreational physical activities. Our study demonstrated that groups of women who practice and do not practice pole dancing differ in terms of education and occupational status. Future studies using linear regression should consider these variables as potential confounders. The recruitment strategy, which utilized snowball sampling and social media groups, further limits the generalizability of this study’s findings. Women with particularly positive attitudes toward pole dancing and those who knew the researcher may overrepresent the sample, unintentionally biasing the results. Future studies should recruit individuals more representative of the general population. The cross-sectional nature of the study precludes causal inferences about the relationships between mind and body. Therefore, future studies should adopt a longitudinal design to ascertain predictive relationships among variables, thereby facilitating the development of intervention programs. Because self-esteem, self-efficacy, and self-acceptance share some common social-cognitive factors, future research could include variables such as perceived social support or perceived autonomy support to control for the extent to which they are related to social impact. Self-report measures are limited by biases, such as social desirability, in which individuals may provide responses that reinforce their self-image rather than reflect the truth. It often leads to overreporting positive behaviors or feelings and underreporting negative ones. Furthermore, issues related to memory and self-awareness can lead to inaccurate data, particularly regarding sensitive topics such as substance use or well-being. Therefore, future research could consider methods of measuring variables other than self-report (e.g., experimental design) to mitigate these limitations. Additionally, cross-cultural research could investigate whether intergroup differences and relationships between variables are culturally specific or universal.

## 5 Conclusion

The study found that participation in pole dancing may have a beneficial effect on self-efficacy and interoceptive awareness, particularly on the “Not Worrying” scale, although the effect sizes for these outcomes were small and further research is needed to confirm this finding. Furthermore, the NA suggests that the key variable for the pole dancing sample is Body Listening. On the other hand, Not-Worrying and Not-Distracting appear to have a negative impact on the network among women who do not participate in pole dancing, suggesting their significant influence relative to other variables in the network. Collectively, these findings can inform coaches that the didactic process should focus on enhancing the ability to read body signals, as well as provide further support for Not Worrying and focusing on Not Distracting during training to improve overall sport performance and well-being. Furthermore, these findings suggest that pole dancing training influences the mind-body network, particularly in relation to interoceptive dimensions such as Body Listening and Not-Worrying. The network analysis also demonstrated a strong interconnection between self-efficacy and self-esteem, both of which are linked to interoception through distinct pathways within the mind-body framework. One primary pathway extends from self-esteem to Noticing, mediated by Trusting, Body Listening, and Emotional Awareness. Another primary pathway extends from self-efficacy to Noticing, mediated by Self-Regulation and Attention Regulation. Given that Noticing represents the most conscious and cognitive aspect of interoception, the study elucidated how pre-reflective bodily experiences are transmitted through specific pathways to distinct dimensions of self-concept, forming a circuit through the Noticing of bodily signals. A secondary, more distal circuit connects self-concept with the Not-Worrying and Not-Distracting dimensions of interoception, underscoring its secondary role in mind-body connections. The network structure identified in this study is corroborated by existing scientific literature. Contemporary neuropsychological and psychosomatic theories, such as the Inference Model, Allostatic Self-Efficacy, and the Psychosomatic Competence Model, can explain it. Consequently, this study contributes to the existing literature by presenting new evidence that supports previous findings and theories on mind-body connections. Additionally, this study may serve as a foundation for designing longitudinal studies to elucidate causal relationships and feedback loops within the mind-body interrelationships, particularly among self-efficacy, self-esteem, and interoceptive awareness.

## Data Availability

The datasets analyzed for this study can be found in the Mendeley Data at the link: https://data.mendeley.com/datasets/df9jys8d9z/1.
