# Correction: Relationships between self-esteem, self-efficacy, and interoceptive awareness among pole dancers: a network analysis

**DOI:** 10.3389/fpsyg.2026.1786390

**Published:** 2026-03-13

**Authors:** Aleksandra M. Rogowska, Magdalena Mikłuszka, Katarzyna Błońska

**Affiliations:** Faculty of Social Sciences, Institute of Psychology, University of Opole, Opole, Poland

**Keywords:** interoceptive awareness, mind-body connections, physical activity, pole dance, self-efficacy, self-esteem, women

In the published article, there was a mistake in the caption of Figure 2. The following last sentence in the caption is incorrect, “The blue line indicates positive associations, and the red line indicates negative associations between nodes.”

The corrected caption of figure 2 appears below.

“Figure 2. Centrality plots for associations between self-esteem, self-efficacy, and interoceptive awareness in the sample of women who do not practice pole dance (red line) and those who practice pole dance (blue line). 1 = Attention regulation, 2 = Body listening, 3 = Emotional awareness, 4 = Not distracting, 5 = Not worrying, 6 = Noticing, 7 = Self-efficacy, 8 = Self-esteem, 9 = Self-regulation, 10 = Trusting.”

The original version of this article has been updated.

